# Efficacy of Metformin in Prevention of Glucocorticoid‐Induced Hyperglycemia in Patients Without Diabetes: A Meta‐Analysis

**DOI:** 10.1002/edm2.70235

**Published:** 2026-06-22

**Authors:** Sana Murtaza, Fatima Murtaza, M. Usama Yasin, Amna Shahid, Maira Shahid, Muhammad Maaz Bin Rehan, Fateen Ata, Muhammad Sohaib Asghar, Mohammed Mahmmoud Fadelallah Eljack

**Affiliations:** ^1^ Punjab Medical College (New: Faisalabad Medical University) Faisalabad Pakistan; ^2^ AdventHealth Sebring Sebring Florida USA; ^3^ Cleveland Clinic Foundation Cleveland Ohio USA; ^4^ Faculty of Medicine and Health Sciences University of Bakhtalruda Ad Douiem Sudan

**Keywords:** glucocorticoids, metabolic complications, metformin, non diabetic

## Abstract

**Purpose:**

The glucocorticoids are increasingly being used in the management of different autoimmune conditions and cancers. Our meta‐analysis aims to leverage metformin's potential to reverse the metabolic complications of glucocorticoids by activating AMP‐activated protein kinase (AMPK), while sparing their anti‐inflammatory benefits.

**Methods:**

PubMed, Cochrane Library, trial registries and Google Scholar were searched from inception to June 2025 for Randomized Controlled Trials (RCTs) and observational studies comparing metformin and placebo to prevent metabolic side effects of glucocorticoid therapy in non‐diabetic patients. After screening by two independent reviewers according to PRISMA guidelines, four studies involving 187 patients were analysed using the random‐effects model in RevMan 5.4.1, and mean differences (MD) were calculated.

**Results:**

Preventive metformin administration during glucocorticoid treatment is superior to placebo with respect to glycaemic control as shown by statistically significant reduction in fasting blood glucose [MD = −0.60 (95% CI: −0.86 to −0.34)], HOMA index of insulin resistance [MD = −0.53 (95% CI: −0.97 to −0.08)], LDL levels [MD = −0.20 (95% CI: −0.40 to −0.00)] and total cholesterol [MD = −0.27 (95% CI: −0.54 to −0.01)]. However, our study failed to show significant improvement in area under the curve glucose (mmol/L × min) after challenge [MD = −176.66 (95% CI: −415.42, 62.11)], basal metabolic rate (BMR) [MD = 51.99 (95% CI: −53.08, 157.07)], HDL [MD = −0.04 (95% CI: −0.13, 0.05)], insulin levels [MD = −2.26 (95% CI: −5.90, 1.38)], triglycerides [MD = −0.15 (95% CI: −0.42, 0.13)] and body weight [MD = −0.93 (95% CI: −2.29 to 0.44)].

**Conclusion:**

While glucocorticoids remain essential in various clinical contexts, their detrimental effects on glucose metabolism are well documented. This study supports metformin as a promising preventive treatment in non‐diabetic patients receiving systemic glucocorticoid therapy. Improvement in insulin sensitivity and resulting fasting glucose levels may offset glucocorticoid‐exacerbated inflammation. Further studies are needed to address long‐term outcomes, including adiposity, bone metabolism, appetite, liver function and muscle wasting, as well as to identify possible predictors of therapeutic response.

**Trial Registration:**

International Prospective Register of Systematic Reviews (PROSPERO) registration number: CRD420251104082

## Graphical abstract

Efficacy of metformin in prevention of glucocorticoid‐induced hyperglycemia in patients without diabetes: Possible preventive role and an avenue worth exploring further.

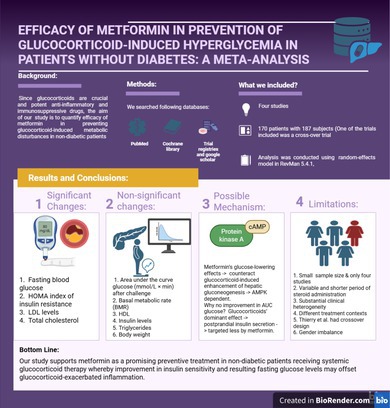

## 1 Introduction

Glucocorticoids (GCs) are potent anti‐inflammatory and immunosuppressive drugs prescribed for a range of medical conditions. The therapeutic efficacy of glucocorticoids (GCs) is well recognized across a range of clinical conditions [1]. However, their use, particularly at high doses, for longer durations has been linked to a range of adverse outcomes. Among these, metabolic complications such as glucose intolerance, dyslipidemia and glucocorticoid‐induced diabetes are especially concerning, even in individuals without pre‐existing metabolic disease [2].

The metabolic disturbances create major life quality problems while simultaneously raising the risk of developing type 2 diabetes mellitus (T2DM) and cardiovascular disease. Glucocorticoid‐induced hyperglycemia has been linked to more extended hospital stays, elevated infection rates and increased mortality [3]. For example, in a retrospective study of 231 patients receiving systemic glucocorticoids for respiratory diseases, 14.7% developed steroid‐induced diabetes, even though they had no prior diagnosis of diabetes. Notably, older age was identified as a significant risk factor for developing glucocorticoid‐induced hyperglycemia, highlighting the clinical importance of early detection and preventive strategies in at‐risk populations [4].

Metformin is a first‐line oral antihyperglycemic agent primarily used in T2DM. It lowers hepatic glucose output, enhances insulin sensitivity and activates AMP‐activated protein kinase (AMPK) [5]. Lipogenic enzymes and gluconeogenic gene transcription are suppressed at the hepatic level by AMPK activation, thereby lowering hepatic glucose production and improving lipid profiles [6]. In clinical studies involving non‐diabetic individuals receiving systemic glucocorticoid therapy, preventive metformin dosing substantially reduced worsening of glycemic indicators and improved metabolic parameters, confirming its potential value in this setting [7].

Metformin may prevent steroid‐induced metabolic disturbances in non‐diabetic populations, but evidence is limited in scale and duration. In a landmark RCT, Seelig et al. [8] found that metformin stabilized glucose AUC levels in 29 non‐diabetic patients, while the placebo group experienced a significant rise over 4 weeks. A crossover study in healthy individuals also confirmed the beneficial action of metformin on insulin sensitivity when exposed to high‐dose prednisone, though the study was conducted for 7 days [1].

Although these findings are promising, they are insufficient to establish metformin as a standard preventive strategy during long‐term glucocorticoid therapy. Therefore, this meta‐analysis aims to systematically evaluate and synthesize the existing evidence regarding the efficacy of metformin in preventing glucocorticoid‐induced metabolic disturbances in non‐diabetic patients.

## 2 Methods

This meta‐analysis is reported following the Preferred Reporting Items for Systematic Reviews and Meta‐Analyses (PRISMA) [9].

### 2.1 Search Strategy

A systematic literature search was conducted across PubMed, the Cochrane Library, trial registries and Google Scholar from inception to June 2025 for Randomized Controlled Trials (RCTs) and observational studies comparing metformin and placebo to prevent metabolic side effects of glucocorticoid therapy in non‐diabetic patients. Other sources used were clinical trial registries and reference list checking. After screening by two independent reviewers according to PRISMA guidelines, four studies involving 170 patients (187 subjects since one of the included trials was a cross‐over trial) were analysed using the random‐effects model in RevMan 5.4.1, and mean differences (MD) were calculated.

### 2.2 Search Terms

The search terms used are (“Metformin”[Mesh] OR “metformin” OR dimethylbiguanide OR Glucophage) AND (“Glucocorticoids”[Mesh] OR “glucocorticoids” OR steroid OR prednisone OR prednisolone OR dexamethasone OR hydrocortisone OR corticosteroid) AND (“Hyperglycemia”[Mesh] OR “hyperglycemia” OR “insulin resistance” OR glucose intolerance OR “metabolic complications” OR “treatment outcome”[Mesh] OR hyperglycaemia OR “Blood Glucose”[MeSH Terms] OR “Insulin” OR high blood glucose OR glucose intolerance OR steroid‐induced diabetes OR glucocorticoid‐induced diabetes OR “Basal Metabolism”[MeSH Terms] OR “Energy Metabolism”[MeSH Terms] OR basal metabolic rate OR BMR OR resting metabolic rate OR RMR OR energy expenditure OR “Body Weight”[MeSH Terms] OR “Body Weight Changes”[MeSH Terms] OR “Body Composition”[MeSH Terms] OR body weight OR body mass OR obesity OR BMI OR body composition OR “Lipids”[MeSH Terms] OR “Cholesterol”[MeSH Terms] OR “Cholesterol, LDL”[MeSH Terms] OR “Cholesterol, HDL”[MeSH Terms] OR “Triglycerides”[MeSH Terms] OR lipid panel OR lipid profile OR cholesterol OR LDL OR HDL OR triglycerides OR total cholesterol OR dyslipidemia OR dyslipidaemia).

### 2.3 Inclusion Criteria

Studies eligible for inclusion needed to meet the following prespecified criteria: (i) studies had to be either randomized controlled trials or observational studies; (ii) participants were adult patients (aged 18 or above) without pre‐existing type 2 diabetes receiving systemic glucocorticoid therapy (prednisone, prednisolone or equivalent); (iii) the intervention group received metformin during glucocorticoid treatment; and (iv) the control group received placebo during glucocorticoid treatment.

### 2.4 Exclusion Criteria

Studies were excluded if (i) they provided reviews of trials that will be published independently or were uncontrolled trials; (ii) they were cross‐sectional studies, commentaries, letters to editors and case reports; (iii) participants had a pre‐existing diagnosis of diabetes mellitus; (iv) participants had prior exposure to metformin shortly before trial enrollment (typically within the last 3 to 6 months); and (v) if they were younger than 18 years of age.

### 2.5 Outcome of Interest

The primary outcomes were (i) hyperglycemia as measured by fasting blood glucose and area under the curve glucose (mmol/L × min) after glucose challenge, (ii) insulin resistance as measured by HOMA index of insulin resistance and insulin levels, (iii) basal metabolic rate (BMR), (iv) body weight, (v) lipid panel including HDL, triglycerides, LDL and total cholesterol.

### 2.6 Data Extraction and Quality Assessment

The articles retrieved from the systematic search were exported to the EndNote Reference Library software and de‐duplicated separately by two reviewers (S.M. and F.M.) (Table 1). The same reviewers then carefully assessed the remaining articles. All trials were initially short‐listed based on title and abstract, after which the complete texts were reviewed to include only those trials that met the previously defined eligibility criteria. Any discrepancies were discussed and resolved through a third reviewer (M.U.). We utilize the PRISMA flow diagram to track the study selection process. A standardized data extraction technique was applied to collect the following information from the eligible studies: (i) author information, (ii) trial phase, (iii) country where the study was conducted, (iv) follow‐up duration, (v) number of participants, (vi) randomized treatment received, (vii) duration of continuous systemic glucocorticoid therapy, (viii) treatment dose and frequency, (ix) age of the participants, (x) sex of the participants, (xi) BMI of the participants, (xii) fasting glucose (mmol/L), (xiii) HOMA2IR levels, (xiv) area under the curve after 75 g sucrose challenge, (xv) triglycerides (mmol/L), (xvi) HDL (mmol/L), (xvii) LDL (mmol/L), (xviii) total cholesterol (mmol/L). Ethical approval was not required, as the study involves secondary data.

**TABLE 1:** Baseline characteristics of studies included.

Study	Thierry et al. [1]	Guinv et al. [10]	Pernicova et al. [11]	Seelig et al. [8]
Year	2025	2020	2020	2017
Randomized treatments	Metformin: 18 Placebo: 18	Metformin: 42 Placebo: 42	Metformin: 26 Placebo: 27	Metformin: 20 Placebo: 14
Centre	University Hospital Basel, Basel, Switzerland	The First Affiliated Hospital of Hainan Medical College, Haikou 570,102, Hainan	Barts Health NHS Trusts	University Hospital Basel and the Cantonal Hospital Aarau
Country	Switzerland	China	UK	Switzerland
Study duration	1 February to 31 August 2021	Jan 2018 to May 2019	July 17, 2012, and Jan 14, 2014	August 2010 to March 2015
Study Design	Single‐center, randomized, placebo‐controlled, double‐blind, crossover trial	Prospective, randomized, single‐blind, single centre clinical study	Randomized, double‐blind, placebo‐controlled proof‐of‐concept study	Randomized, placebo‐controlled, double‐blind study
NCT number	NCT04659915	N/A	NCT01319994	NCT01187849
Trial phase	Phase 4	N/A	Phase 2	Phase 4
Number of original participants	18	84	53	34
Duration of treatment	Two 7‐day periods separated by a 28‐day washout period	1 month	12 weeks	4 weeks
Dose and frequency	Prednisone 30 mg/day, two 7‐day periods with 28‐day washout Metformin500–2000 mg/day over 7 days (titrated every other day by 500 mg) Placebo Matching 7‐day period	Metformin 500 mg TDS + prednisone 40 mg per day Placebo TDS + prednisone 40 mg per day	Metformin: 850 mg TDS: 850 mg/day for the first 5 days, 850 mg twice a day for the next 5 days and 850 mg three times a day subsequently Placebo: TDS with similar 850 mg/day for the first 5 days, 850 mg twice a day for the next 5 days and 850 mg three times a day subsequently	850 mg od × 1 week, then 850 mg BID × 3 weeks 850 mg od × 1 week, then 850 mg BID × 3 weeks
Age (years)	27 ± 5.19	37 ± 8 37 ± 8	47 ± 15 45 ± 15	58.0 (35.8–74.3) 56.5 (46.5–67.8)
Sex (M/F)	18/0	9/31 7/33	12/14 12/15	14/6 5/9
BMI	22.85 ± 1.84	21.7 ± 1.2 22.2 ± 1.2	27.3 (23.3–36.1) 28.5 (24.7–35.8)	24.2 (21.6–28.6) 25.7 (20.6–27.5)
Fasting glucose (mmol/L)	4.56 ± 0.26	N/A	4.7 (4.5–5.7) 4.9 (4.5–5.8)	4.8 (4.6–5.3) 5.0 (4.6–5.3)
HOMA2IR	N/A	N/A	4.7 (2.2–5.5) 4.1 (2.8–5.4)	1.9 (1.0–3.4) 1.0 (0.5–2.0)
Area under the curve glucose (mmol/L × min) after 75 g sucrose challenge	N/A	N/A	826 (643–1054) 806 (717–1179)	937.5 (872.3–991.1) 864.8 (782.6–1012.1)
Triglycerides (mmol/L)	0.96 ± 0.37	N/A	1.5 (1.0–2.3) 1.3 (1.1–1.8)	1.3 (0.9–1.7) 1.1 (0.9–1.2)
HDL Cholesterol (mmol/L)	1.28 ± 0.32	N/A	1.86 (0.52) 1.81 (0.46)	1.2 (1.0–1.4) 1.4 (1.0–1.7)
LDL Cholesterol (mmol/L)	2.28 ± 0.95	N/A	2.8 (2.3–3.4) 2.9 (2.5–3.4)	3.1 (2.5–3.8) 2.9 (2.6–3.1)
Total Cholesterol (mmol/L)	4.00 ± 1.00	N/A	5.6 (4.7–6.4) 5.4 (4.7–6.2)	4.8 (4.3–5.6) 4.8 (4.4–5.2)

### 2.7 Data Synthesis

We synthesized the data for the following outcomes (i) Area under the curve glucose (mmol/L × min) after 75 g sucrose challenge, (ii) HOMA index, (iii) fasting blood glucose levels, (iv) weight, (v) insulin levels, (vi) BMR, (vii) LDL cholesterol levels, (viii) HDL cholesterol levels, (ix) total cholesterol levels and (x) triglyceride levels.

The statistical analysis was performed using Review Manager (RevMan, Version 5.4.1) software. For the primary outcome of all‐cause mortality, raw data were pooled to calculate mean differences with 95% confidence intervals (CIs), using a random‐effects model to account for anticipated heterogeneity across studies. Heterogeneity among the studies was assessed using the Higgins *I* ^2^ index and the Chi^2^ tests [12].

Given the limited number of studies, traditional funnel plot analysis for publication bias was impractical; thus, the Doi plot was used as an alternative graphical method. The Doi plot compares the outcome of interest against the absolute value of the *z*‐score from the studies, providing a more accurate estimation of the asymmetry.

### 2.8 Subgroup and Sensitivity Analysis

Subgroup and sensitivity analyses were conducted to explore the observed heterogeneity. The Higgins *I* ^2^ statistic was employed, with *I* ^2^ values below 50% considered acceptable. Statistical significance was determined at a *p*‐value < 0.05 (Figures S3.1–S3.4).

### 2.9 Risk of Bias Assessment

To assess the quality of the included randomized controlled trials (RCTs), we used the Cochrane risk‐of‐bias 2 (RoB 2) tool [13]. The studies were analysed based on domains including the randomization process, the timing of participant identification or recruitment, deviations from the intended interventions, missing outcome data, measurement of the outcome and selection of the reported result. Methodological components of the studies were assessed and classified into high‐, low‐ and unclear‐risk categories. Two independent reviewers (S.M. and F.M.) evaluated the risk of bias for all the eligible trials using this tool. Discrepancies were discussed and clarified with the third author (M.U.) to make a final recommendation.

### 2.10 Quality of Evidence Assessment

The quality of evidence was assessed per outcome, independently by two individuals (SM and FM), using the GRADE guidelines for rating quality of evidence [14, 15]. The Grading of Recommendations Assessment, Development and Evaluation (GRADE) method was used to assess the strength of the evidence for each outcome. Key findings and corresponding levels of certainty will be presented in a summary of findings table. The quality of evidence for each outcome was rated as high, moderate, low or very low.

## 3 Results

### 3.1 Study Selection

**FIGURE 1:**
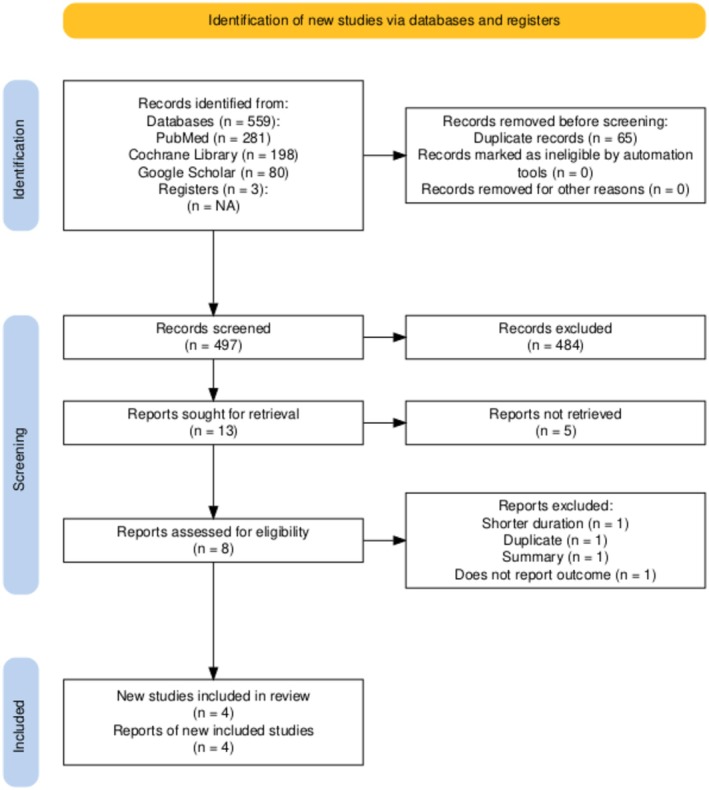
PRISMA flow diagram. *Source:* Page MJ et al. *BMJ* (2021), 372: n71. doi: https://doi.org/10.1136/bmj.n71.

A flow diagram of literature retrieval is shown in the Figure 1. A total of 562 studies were retrieved, including 281 from PubMed, three from trial registries, 80 from Google Scholar and 198 from the Cochrane Library. Sixty‐five were removed due to duplication, and 484 were excluded during preliminary screening. The remaining 13 studies were thereafter screened for full‐text assessment. In this process, five reports were not retrieved. The remaining eight studies were assessed for eligibility, of which one was excluded because it had a shorter duration, one because it was a duplicate study, one because it was a summary, and one because it didn't report the outcome. Finally, four studies were included in this meta‐analysis [16].

### 3.2 Study Characteristics

There were 170 patients (187 subjects since one was a crossover trial) from four studies. The included studies consisted of 4 RCTs. The participants were predominantly female (152), all older than 18 years of age. The minimum number of participants in an individual study was 17, and the maximum was 84. Regarding the study area, participants were from China, Switzerland and the UK.

### 3.3 Statistical Analysis

The effect of metformin on glucocorticoid‐induced metabolic disturbances was expressed in terms of the following variables.

#### 3.3.1 AUC Under the Curve Glucose

According to our analysis of 3 RCTs, the pooled mean difference favoured metformin but was not statistically significant (mean difference: −176.66; 95% CI: −415.42 to 62.11; *p* = 0.15). There was considerable heterogeneity among the included studies (*I* ^2^ = 96%, Tau^2^ = 42258.97, Chi^2^ = 50.48, df = 2; *p* < 0.00001), indicating variability in study effects when metformin was utilized compared to a placebo. The GRADE evaluation yielded very low‐quality evidence synthesis for the outcome (Figure S2.1, Table S1).

#### 3.3.2 Fasting Blood Glucose

**FIGURE 2:**
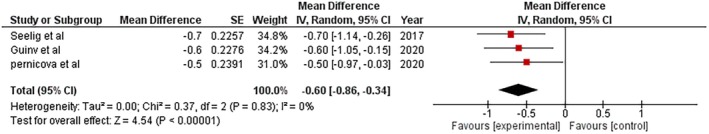
Forest plot showing the pooled effect of metformin on Fasting Blood Glucose.

According to our analysis of 3 RCTs, the pooled mean difference was −0.60 (95% CI: −0.86 to −0.34; *p* < 0.00001), indicating a statistically significant difference between the metformin and placebo groups. No heterogeneity was observed among studies (*I* ^2^ = 0%, Tau^2^ = 0.00, Chi^2^ = 0.37, df = 2; *p* = 0.83), suggesting consistent findings across trials when metformin was used compared to placebo. The GRADE evaluation yielded a high‐quality evidence synthesis for the outcome (Figure 2, Table S1).

#### 3.3.3 Insulin Levels

According to our analysis of 2 RCTs, the pooled mean difference was −2.26 (95% CI: −5.90 to 1.38; *p* = 0.22), indicating no statistically significant difference between the metformin and placebo groups. There was substantial heterogeneity between the studies (*I* ^2^ = 62%, Tau^2^ = 4.96, Chi^2^ = 2.63, df = 1; *p* = 0.10), indicating variability in study effects when metformin was utilized compared to a placebo. The GRADE evaluation yielded a moderate‐quality evidence synthesis for the outcome (Figure S2.2, Table S1).

#### 3.3.4 HOMA Index

**FIGURE 3:**
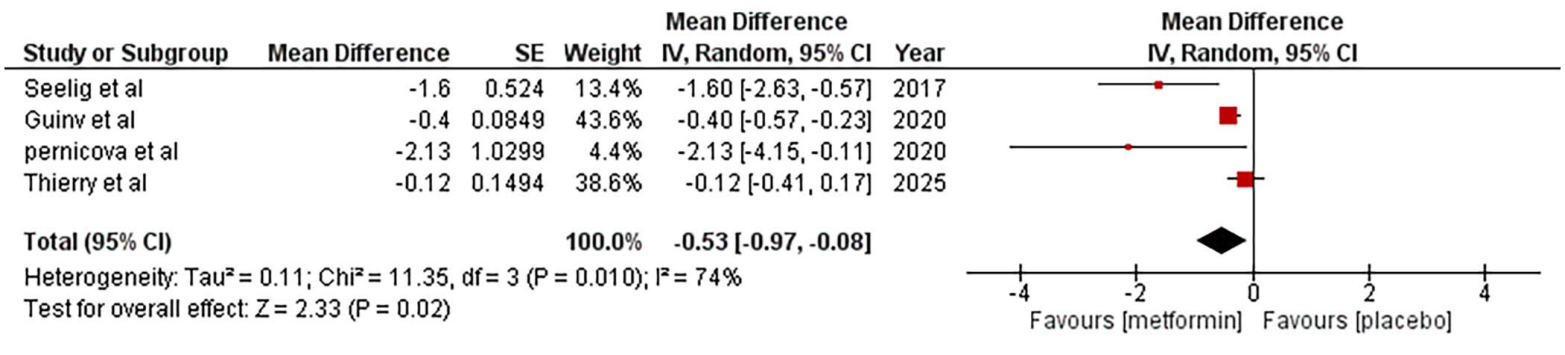
Forest plot showing the pooled effect of metformin on the HOMA Index.

According to our analysis of 4 RCTs, the pooled mean difference was −0.53 (95% CI: −0.97 to −0.08; *p* = 0.02), indicating a statistically significant difference between the metformin and placebo groups. There was substantial heterogeneity between the studies (*I* ^2^ = 74%, Tau^2^ = 0.11, Chi^2^ = 11.35, df = 3; *p* = 0.010), indicating variability in study effects when metformin was utilized compared to placebo. The GRADE evaluation yielded moderate‐quality evidence synthesis for the outcome (Figure 3, Table S1).

#### 3.3.5 LDL

According to our analysis of 3 RCTs, the pooled mean difference was −0.20 (95% CI: −0.40 to −0.00; *p* = 0.05), indicating a statistically significant difference between the metformin and placebo groups. There was no heterogeneity among the studies (*I* ^2^ = 0%, Tau^2^ = 0.00, Chi^2^ = 1.31, df = 2; *p* = 0.52), suggesting consistent findings across trials when metformin was used compared to placebo. The GRADE evaluation yielded a moderate‐quality evidence synthesis for the outcome (Figure S2.3, Table S1).

#### 3.3.6 HDL

According to our analysis of 2 RCTs, there was a pooled mean difference of −0.04 (95% CI: −0.13 to 0.05; *p* < 0.37), indicating no statistically significant difference between the metformin and placebo groups. No heterogeneity was observed among the studies (*I* ^2^ = 0%, Tau^2^ = 0.00, Chi^2^ = 0.03, df = 1; *p* = 0.86), suggesting consistent findings across trials when metformin was used compared to placebo. The GRADE evaluation yielded moderate‐quality evidence synthesis for the outcome (Figure S2.4 and Table S1).

#### 3.3.7 Triglycerides

According to our analysis of 2 RCTs, the pooled mean difference was −0.15 (95% CI: −0.42 to 0.13; *p* = 0.30), indicating no statistically significant difference between the metformin and placebo groups. There was no heterogeneity among the studies (*I* ^2^ = 0%, Tau^2^ = 0.00, Chi^2^ = 0.07, df = 1; *p* = 0.79), suggesting consistent findings across trials when metformin was used compared to placebo. The GRADE evaluation yielded moderate‐quality evidence synthesis for the outcome (Figure S2.5, Table S1).

#### 3.3.8 Total Cholesterol

According to our analysis of 2 RCTs, the pooled mean difference was −0.27 (95% CI: −0.54 to −0.01; *p* = 0.05), indicating a statistically significant difference between the metformin and placebo groups. There was no heterogeneity among the studies (*I* ^2^ = 0%, Tau^2^ = 0.00, Chi^2^ = 0.02, df = 1; *p* = 0.87), suggesting consistent findings across trials when metformin was used compared to placebo. The GRADE evaluation yielded a high‐quality evidence synthesis for the outcome (Figure S2.6 and Table S1).

#### 3.3.9 Weight

According to our analysis of 2 RCTs, the pooled mean difference was −0.93 (95% CI: −2.29 to 0.44; *p* = 0.18), indicating no statistically significant difference between the metformin and placebo groups. There was considerable heterogeneity between the studies (*I* ^2^ = 92%, Tau^2^ = 0.89, Chi^2^ = 12.89, df = 1; *p* = 0.0003), indicating variability in study effects when metformin was utilized compared to a placebo. The GRADE evaluation yielded a low‐quality evidence synthesis for the outcome (Figure S2.7 and Table S1).

### 3.4 BMR

According to our analysis of 2 RCTs, the pooled mean difference was 51.99 (95% CI: −53.08 to 157.07; *p* = 0.33), indicating no statistically significant difference between the metformin and placebo groups. No heterogeneity was observed among studies (*I* ^2^ = 0%, Tau^2^ = 0.00, Chi^2^ = 0.01, df = 1; *p* = 0.91), suggesting consistent findings across trials when metformin was used compared to placebo. The GRADE evaluation yielded a low‐quality evidence synthesis for the outcome (Figure S2.8, Table S1).

### 3.5 Heterogeneity

The heterogeneity of included studies was substantial for the outcomes Glucose Area Under the Curve (AUC) (*I* ^2^ = 96%), insulin levels (*I* ^2^ = 62%), HOMA Index (*I* ^2^ = 74%) and weight (*I* ^2^ = 92%), as shown in the figure below. The substantial *I* ^2^ can be partly explained by the fact that Thierry et al. [1] was a crossover trial. Moreover, a regression analysis was not performed as it would have further reduced the number of studies included in the meta‐analysis.

### 3.6 Sensitivity Analysis

To explore sources of heterogeneity, we conducted multiple sensitivity analyses. Sequential removal of individual studies did not materially alter the pooled effect estimate (Figures S3.1–S3.4).

### 3.7 Measures of Bias

**FIGURE 4:**
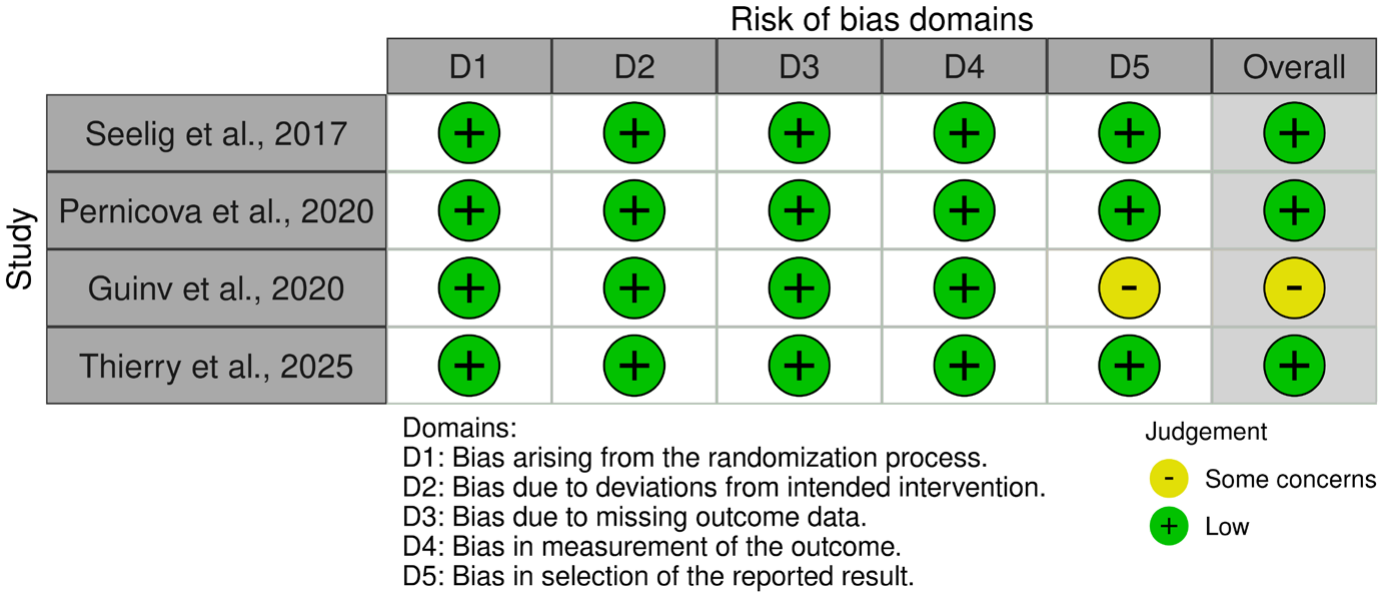
Traffic light plot of risk of bias assessment of RCTs.

We assessed the risk of bias using the Cochrane risk of bias tool across several domains, including the randomization process, deviations from intended interventions, missing outcome data, the outcome measurement and selection of the reported results. The analysis indicated that all but one study had a low risk of bias in all assessed domains, as shown in the summary plot of the risk of bias assessment (Figures S1.1 and S1.2). Specifically, all but Guinv et al.'s [10] study achieved 100% low risk for the randomization process, deviations from intended interventions, missing outcome data, measurement of the outcome, and selection of the reported result, indicating robust and reliable methodologies across the trials. The studies consistently focused on metformin as an intervention. The overall uniform low risk of bias across all domains further strengthens the validity of the meta‐analysis conclusions. The traffic light plot of the risk of bias is shown in Figure 4.

The Grade Analysis of 4 studies on total cholesterol and fasting blood glucose demonstrated high certainty of evidence. On the other hand, the area under the glucose curve (mmol/L × min) after the 75 g sucrose challenge demonstrated a very low level of certainty of evidence; differences in weight and BMR demonstrated a low level of certainty of evidence; and HDL cholesterol, the HOMA index, triglyceride, LDL and insulin levels showed a moderate level of certainty of evidence, respectively [17].

### 3.8 Publication Bias

Doi plots for assessment of asymmetry are presented in Figures S1.1.1–1.1.4. The LFK indices were −10.83 for AUC after glucose challenge test (major asymmetry), 0.11 for fasting blood glucose (no asymmetry), −5.82 for HOMA Index (major asymmetry) and −2.19 for LDL levels (major asymmetry). However, given the small number of included studies per outcome, high heterogeneity in some analyses and known instability of the LFK index in meta‐analyses with few studies (Schwarzer et al. [18]), these findings should be interpreted cautiously and do not provide strong evidence of publication bias.

## 4 Discussion

Glucocorticoids with powerful anti‐inflammatory and immunosuppressive effects remain indispensable for managing a broad spectrum of conditions, including inflammatory, autoimmune and allergic conditions, as well as preventing transplant rejection and stabilizing critically ill patients. However, glucocorticoid‐induced hyperglycemia poses significant clinical challenges. A meta‐analysis by Liu et al. [19] revealed that 32.3% of patients receiving glucocorticoid therapy develop steroid‐induced hyperglycemia, while 18.6% progress to diabetes mellitus. Given metformin's well‐documented efficacy in type 2 diabetes, its potential application for preventing glucocorticoid‐induced metabolic disturbances appears mechanistically justified.

This meta‐analysis of 4 randomized controlled trials (*n* = 187) provides the first pooled evidence that prophylactic metformin significantly mitigates several glucocorticoid‐induced metabolic abnormalities in non‐diabetic adults. Our findings demonstrate clinically meaningful reductions in fasting glucose [MD = −0.60, 95% CI: −0.86 to −0.34], improvements in insulin sensitivity as indicated by a decrease in HOMA‐IR [MD = −0.53, 95% CI: −0.97 to −0.08], and modest reductions in atherogenic lipids, including LDL levels [MD = −0.20, 95% CI: −0.40 to −0.00] and total cholesterol [MD = −0.27, 95% CI: −0.54 to −0.01]. However, it showed no benefit for post‐challenge glycemia, body weight, HDL, triglycerides and basal metabolic rate.

Metformin's glucose‐lowering effects likely result from its ability to counteract glucocorticoid‐induced enhancement of hepatic gluconeogenesis, mediated by increased expression of PEPCK and G6Pase, and to improve insulin signalling in peripheral tissues [13]. These actions are largely AMPK‐dependent and include suppression of hepatic gluconeogenesis, inhibition of mitochondrial glycerol‐3‐phosphate dehydrogenase (mG3PDH), and protection of pancreatic β‐cells through reduction of endoplasmic reticulum (ER) stress and prevention of calcium‐induced mitochondrial permeability transition pore (PTP) opening [20]. This holds clinical significance, as elevated blood glucose is linked to adverse outcomes, including increased cardiovascular risk, even in non‐diabetic individuals [21]. The lack of improvement in AUC glucose may reflect glucocorticoids' dominant disruption of postprandial insulin secretion, a pathway less targeted by metformin.

Our findings of reduced LDL and total cholesterol levels suggest that, beyond its role in glycemic control, metformin exerts an antagonistic effect on glucocorticoid‐induced dyslipidemia. This effect may be attributed to metformin's activation of AMPK, which inhibits SREBP1c, which is a key regulator of lipid synthesis. This inhibition downregulates enzymes like FADS1 and FADS2, reducing hepatic lipid production and potentially lowering LDL‐C levels [22]. The neutral impact on weight and BMR suggests metformin does not offset glucocorticoid‐induced muscle catabolism, possibly due to short study duration or limited sample size. Longer treatment may reveal different outcomes.

### 4.1 Strengths and Limitations

To our knowledge, this is the first study to assess the preventive use of metformin for glucocorticoid‐induced metabolic complications specifically in non‐diabetic adults. We followed PRISMA guidelines and used the GRADE approach to ensure high‐quality evidence. To reduce selection bias, two independent reviewers handled study selection and data extraction. Despite notable heterogeneity, our primary outcomes remained consistent across sensitivity analyses, supporting the reliability of the findings.

This meta‐analysis has several significant limitations. First, the pooled sample was small (*n* = 187) and comprised only four studies, reducing statistical power, limiting the scope for subgroup analyses and making the results more sensitive to outliers. Second, a future analysis should include any steroid duration with proper sub‐grouping. Ironically, all trials featured short follow‐up periods (≤ 12 weeks), which prevented the assessment of critical longer‐term outcomes such as diabetes incidence or cardiovascular risk. Third, although substantial clinical heterogeneity was observed across studies, we were unable to adequately examine its sources because key effect modifiers were inconsistently reported. Fourth, although sensitivity analyses confirmed the robustness of our primary findings, they revealed a paradoxical increase in heterogeneity (*I* ^2^ = 85%–88%) upon sequential exclusion of individual studies. This pattern should not be dismissed as mere statistical noise; instead, it suggests that metformin's effects may be modified by patient characteristics or treatment context. Possible explanations include carryover effects from Thierry et al.'s [1] crossover design and the markedly different exposure profile created by Pernicova et al.'s extended tapering schedule, in contrast to the fixed 4‐week regimens used in other trials. Such variability intrinsically reflects real‐world clinical practice and underscores the need for tailored metformin strategies aligned with specific steroid treatment patterns. Finally, the pronounced gender imbalance, with 89% of participants female, substantially limits generalizability to male populations, particularly given known sex differences in glucocorticoid metabolism and metformin pharmacokinetics.

## Author Contributions

**Sana Murtaza:** conceptualization, writing – original draft, visualization, writing – review and editing. **Muhammad Maaz Bin Rehan:** writing – review and editing, methodology, software, resources. **Amna Shahid:** conceptualization, visualization, investigation, resources. **Fatima Murtaza:** conceptualization, data curation, writing – original draft. **M. Usama Yasin:** conceptualization, writing – original draft, software. **Maira Shahid:** conceptualization, data curation, validation, investigation. **Muhammad Sohaib Asghar:** conceptualization, writing – review and editing, project administration, supervision, formal analysis. **Fateen Ata:** conceptualization, writing – review and editing, visualization, validation. **Mohammed Mahmmoud Fadelallah Eljack:** writing – review and editing, project administration, supervision.

## Funding

The authors have nothing to report.

## Ethics Statement

The authors have nothing to report.

## Conflicts of Interest

The authors declare no conflicts of interest.

## Supporting information


**Data S1:** edm270235‐sup‐0001‐FigureS1‐S3.docx.
**Figure S1:1.1:** Doi Plot for AUC after glucose challenge test.
**Figure S1:1.2:** DOI plot for fasting blood glucose.
**Figure S1:1.3:** DOI plot for HOMA Index.
**Figure S1:1.4:** DOI plot for LDL levels.
**Figure S1:2:** Summary Plot of Risk of Bias Assessment for RCTs.
**Figure S2:1:** Forest plot showing pooled effect of metformin on Glucose Area Under the Curve (AUC).
**Figure S2:2:** Forest plot showing pooled effect of metformin on insulin levels.
**Figure S2:3:** Forest plot showing pooled effect of metformin on LDL cholesterol.
**Figure S2:4:** Forest plot showing pooled effect of metformin on HDL cholesterol.
**Figure S2:5:** Forest plot showing pooled effect of metformin on Triglycerides levels.
**Figure S2:6:** Forest plot showing pooled effect of metformin on Total cholesterol.
**Figure S2:7:** Forest plot showing pooled effect of metformin on Weight.
**Figure S2:8:** Forest plot showing pooled effect of metformin on Basal Metabolic Rate (BMR).
**Figure S3:1:** Sensitivity analysis of AUC after glucose challenge after removing Pernicovola et al. study.
**Figure S3:2:** Sensitivity analysis of AUC after glucose challenge after removing Seelig et al. study.
**Figure S3:3:** Sensitivity analysis of AUC after glucose challenge after removing Thierry et al. study.
**Figure S3:4:** Sensitivity analysis of HOMA index after removing Seelig et al. study.
**Figure S4:** Summary of findings including GRADE Assessment.

## Data Availability

The data that support the findings of this study are available from the corresponding author upon reasonable request.
