# Supplementary material for: Efficacy of Metformin in Prevention of Glucocorticoid‐Induced Hyperglycemia in Patients Without Diabetes: A Meta‐Analysis
Source: Endocrinol Diabetes Metab. 2026 Jun 22;9(4):e70235. doi: 10.1002/edm2.70235 (PMC13287083; doi:10.1002/edm2.70235)
Supplement: Supplementary file 1 — Data S1: edm270235‐sup‐0001‐FigureS1‐S3.docx. Figure S1:1.1: Doi Plot for AUC after glucose challenge test. Figure S1:1.2: DOI plot for fasting blood glucose. Figure S1:1.3: DOI plot for HOMA Index. Figure S1:1.4: DOI plot for LDL levels. Figure S1:2: Summary Plot of Risk of Bias Assessment for RCTs. Figure S2:1: Forest plot showing pooled effect of metformin on Glucose Area Under the Curve (AUC). Figure S2:2: Forest plot showing pooled effect of metformin on insulin levels. Figure S2:3: Forest plot showing pooled effect of metformin on LDL cholesterol. Figure S2:4: Forest plot showing pooled effect of metformin on HDL cholesterol. Figure S2:5: Forest plot showing pooled effect of metformin on Triglycerides levels. Figure S2:6: Forest plot showing pooled effect of metformin on Total cholesterol. Figure S2:7: Forest plot showing pooled effect of metformin on Weight. Figure S2:8: Forest plot showing pooled effect of metformin on Basal Metabolic Rate (BMR). Figure S3:1: Sensitivity analysis of AUC after glucose challenge after removing Pernicovola et al. study. Figure S3:2: Sensitivity analysis of AUC after glucose challenge after removing Seelig et al. study. Figure S3:3: Sensitivity analysis of AUC after glucose challenge after removing Thierry et al. study. Figure S3:4: Sensitivity analysis of HOMA index after removing Seelig et al. study. Figure S4: Summary of findings including GRADE Assessment. [file EDM2-9-e70235-s001.docx]

#### **Supplementary Information**

#### **Article Title:** EFFICACY OF METFORMIN IN PREVENTION OF GLUCOCORTICOID-INDUCED HYPERGLYCEMIA IN PATIENTS WITHOUT DIABETES: A META-ANALYSIS

1. **Measures of bias:**

**1.1:DOI Plots**:

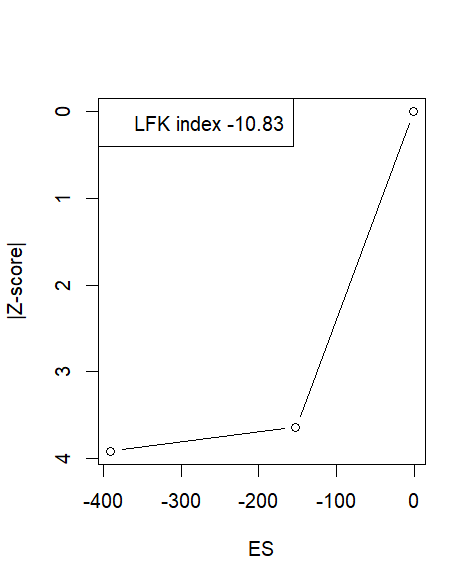


**Supplementary Figure 1.1.1:** Doi Plot for AUC after glucose challenge test


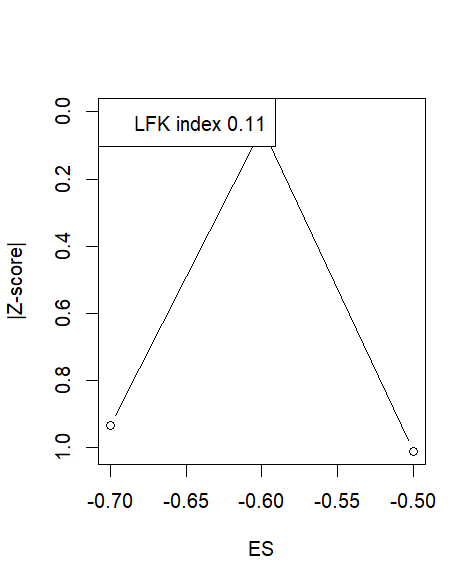

**Supplementary Figure 1.1.2:** DOI plot for fasting blood glucose


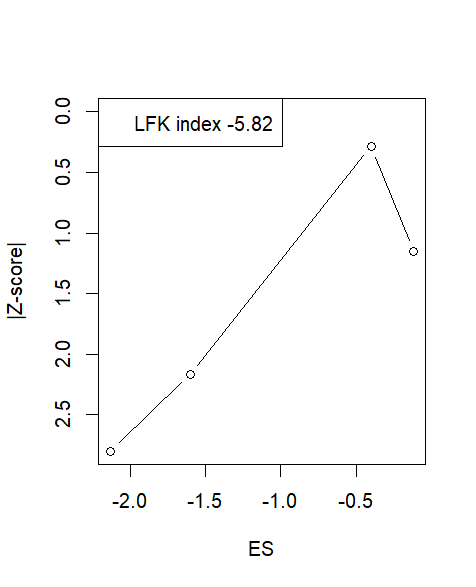


**Supplementary Figure 1.1.3: DOI plot for HOMA Index**


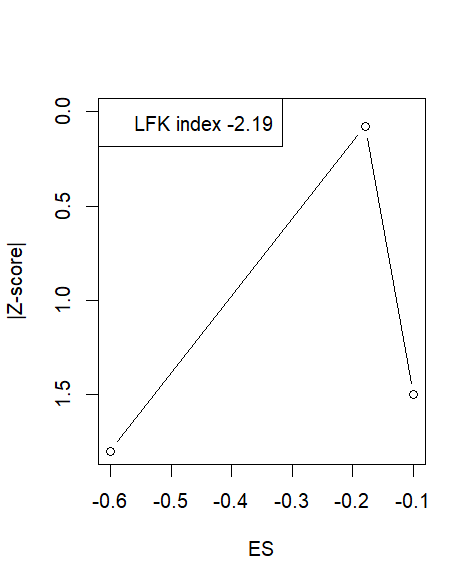


**Supplementary Figure 1.1.4:** DOI plot for LDL levels

**1.2 Risk of bias assessment:**


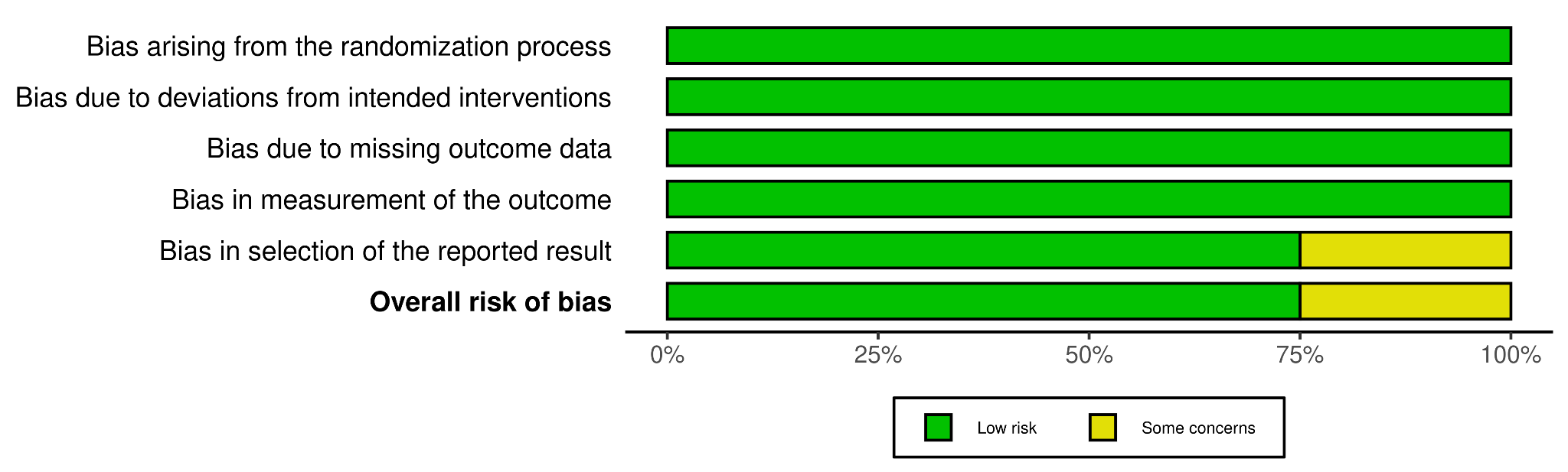


#### **Supplementary Figure 1.2:** Summary Plot of Risk of Bias Assessment for RCTs

**2. Pooled Estimates and Forest Plots:**


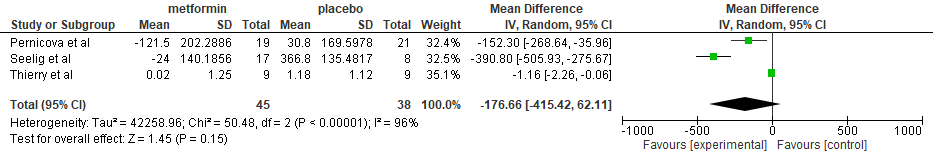

**Supplementary Figure 2.1:** Forest plot showing pooled effect of metformin on Glucose Area Under the Curve (AUC)

**
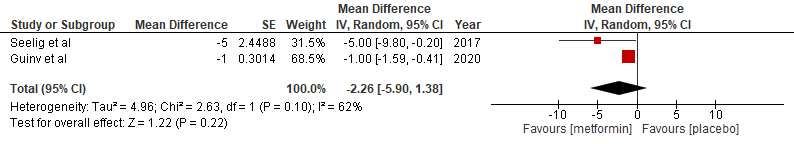
**

**Supplementary Figure 2.2:** Forest plot showing pooled effect of metformin on insulin levels

**
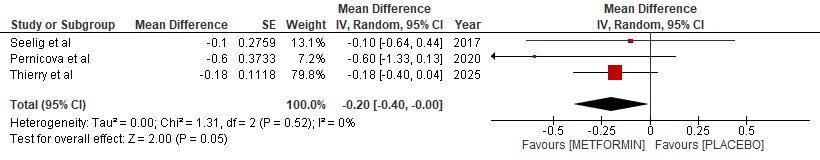
**

**Supplementary Figure 2.3:** Forest plot showing pooled effect of metformin on LDL cholesterol

**
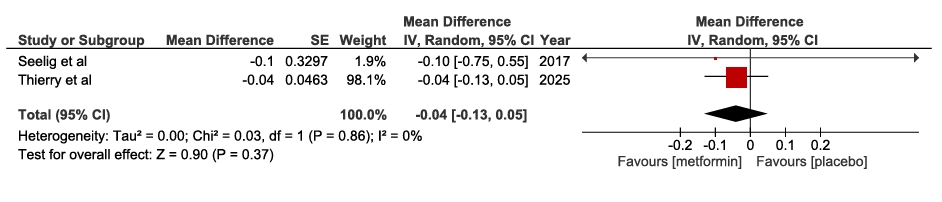
**

**Supplementary Figure 2.4:** Forest plot showing pooled effect of metformin on HDL cholesterol

**
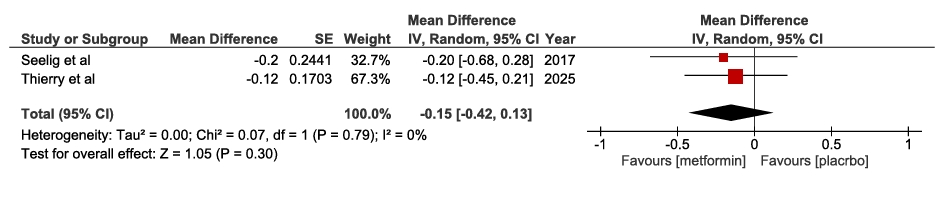
**

**Supplementary Figure 2.5:** Forest plot showing pooled effect of metformin on Triglycerides levels

**
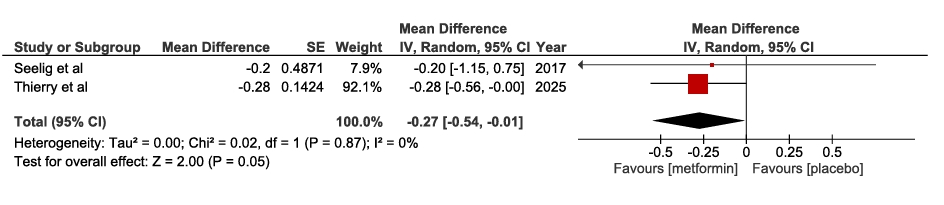
**

**Supplementary Figure 2.6:** Forest plot showing pooled effect of metformin on Total cholesterol

**
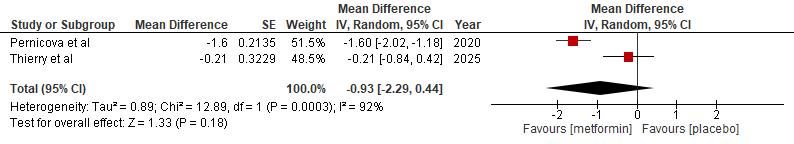

Supplementary Figure 2.7:** Forest plot showing pooled effect of metformin on Weight

**
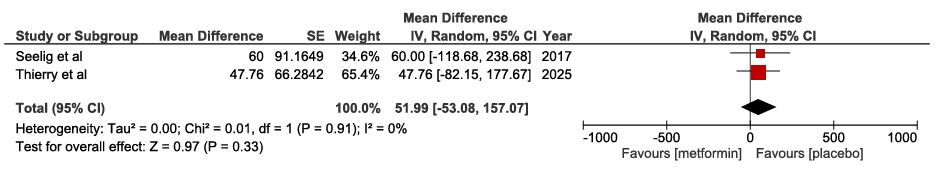

Supplementary Figure 2.8:** Forest plot showing pooled effect of metformin on Basal Metabolic Rate (BMR)

**3. Sensitivity analyses:**


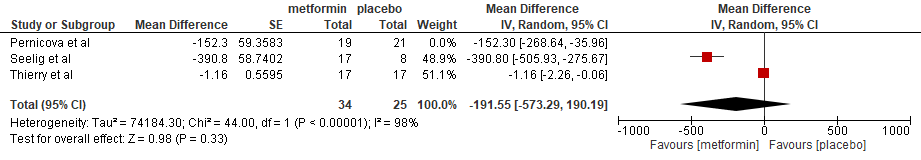


**Supplementary Figure 3.1:** Sensitivity analysis of AUC after glucose challenge after removing Pernicovola et al. study


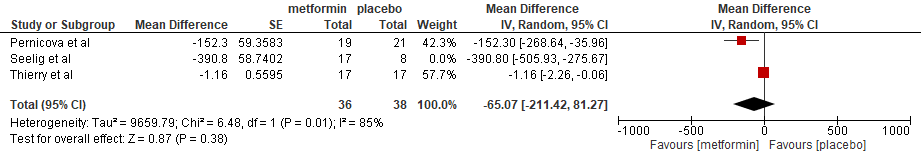


**Supplementary Figure 3.2:** Sensitivity analysis of AUC after glucose challenge after removing Seelig et al. study


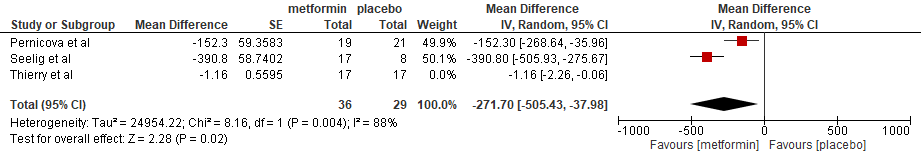


**Supplementary Figure 3.3:** Sensitivity analysis of AUC after glucose challenge after removing Thierry et al. study


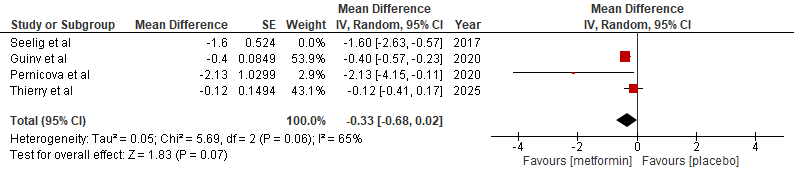


**Supplementary Figure 3.4:** Sensitivity analysis of HOMA index after removing Seelig et al. study

**4. Summary of findings including GRADE Assessment:**

| **Certainty assessment** | | | | | | | **№ of patients** | | **Effect** | | **Certainty** | **Importance** |
| --- | --- | --- | --- | --- | --- | --- | --- | --- | --- | --- | --- | --- |
| **№ of studies** | **Study design** | **Risk of bias** | **Inconsistency** | **Indirectness** | **Imprecision** | **Other considerations** | **Metformin** | **placebo** | **Relative  (95% CI)** | **Absolute  (95% CI)** |  |  |
| **Area under the curve glucose (mmol/L × min) after 75 g sucrose challenge** | | | | | | | | | | | | |
| 3 | randomised trials | not serious | serious^a^ | not serious | very serious^b^ | publication bias strongly suspected^c^ | 53 | 46 | - | MD **176.66 lower**  (415.42 lower to 62.11 higher) | ⨁◯◯◯  Very low^a,b,c^ | CRITICAL |
| **HOMA index** | | | | | | | | | | | | |
| 4 | randomised trials | not serious | not serious | not serious | not serious | publication bias strongly suspected^d^ | 0 | 0 | - | MD **0.53 lower**  (0.97 lower to 0.08 lower) | ⨁⨁⨁◯  Moderate^d^ | CRITICAL |
| **Fasting blood glucose** | | | | | | | | | | | | |
| 3 | randomised trials | not serious | not serious | not serious | not serious | none | 78 | 74 | - | MD **0.6 lower**  (0.86 lower to 0.34 lower) | ⨁⨁⨁⨁  High | CRITICAL |
| **Weight** | | | | | | | | | | | | |
| 2 | randomised trials | not serious | serious^e^ | not serious | serious^f^ | none | 0 | 0 | - | MD **0.93 lower**  (2.29 lower to 0.44 higher) | ⨁⨁◯◯  Low^e,f^ | IMPORTANT |
| **Insulin levels** | | | | | | | | | | | | |
| 2 | randomised trials | not serious | not serious | not serious | serious^g^ | none | 0 | 0 | - | MD **2.26 lower**  (5.9 lower to 1.38 higher) | ⨁⨁⨁◯  Moderate^g^ | CRITICAL |
| **BMR** | | | | | | | | | | | | |
| 2 | randomised trials | not serious | not serious | not serious | very serious^h^ | none | 0 | 0 | - | MD **51.99 higher**  (53.08 lower to 157.07 higher) | ⨁⨁◯◯  Low^h^ | IMPORTANT |
| **LDL** | | | | | | | | | | | | |
| 3 | randomised trials | not serious | not serious | not serious | not serious | publication bias strongly suspected^i^ | 0 | 0 | - | MD **0.2 lower**  (0.4 lower to 0 ) | ⨁⨁⨁◯  Moderate^i^ | IMPORTANT |
| **Total cholesterol** | | | | | | | | | | | | |
| 2 | randomised trials | not serious | not serious | not serious | not serious | none | 0 | 0 | - | MD **0.27 lower**  (0.54 lower to 0.01 lower) | ⨁⨁⨁⨁  High | IMPORTANT |
| **HDL cholesterol** | | | | | | | | | | | | |
| 2 | randomised trials | not serious | not serious | not serious | serious^j^ | none | 0 | 0 | - | MD **0.04 lower**  (0.13 lower to 0.05 higher) | ⨁⨁⨁◯  Moderate^j^ | IMPORTANT |
| **Triglycerides** | | | | | | | | | | | | |
| 2 | randomised trials | not serious | not serious | not serious | serious^k^ | none | 0 | 0 | - | MD **0.15 lower**  (0.42 lower to 0.13 higher) | ⨁⨁⨁◯  Moderate^k^ | IMPORTANT |

**CI:** confidence interval; **MD:** mean difference

#### **Explanations**

a. High heterogeneity observed across studies (I² = 96%) with statistically significant Chi² (P < 0.00001) and a large Tau² (42,258.97).

b. Wide 95% Confidence intervals (-415.42, to +62.11) and small sample size.

c. The Doi plot demonstrated major asymmetry (LFK Index= -10.83), suggesting small-study effects but the possibility of publication bias cannot be ruled out.

d. The Doi plot demonstrated major asymmetry (LFK Index= -5.82), suggesting small-study effects but the possibility of publication bias cannot be ruled out.

e. High heterogeneity ((I² = 92%) observed across studies with statistically significant Chi² (P = 0.0003).

f. Wide 95% confidence intervals (-2.29, to 0.44) and small sample size.

g. Wide 95% confidence intervals (-5.90, to 1.38) and small sample size.

h. Wide 95% confidence intervals (-53.08 to 157.07) and small sample size.

i. The Doi plot demonstrated somewhat major asymmetry (LFK Index= -2.19), suggesting small-study effects but the possibility of publication bias cannot be ruled out.

j. Wide 95% confidence intervals (-0.13, 0.05) and small sample size.

k. Wide 95% confidence intervals (-0.42, 0.13) and small sample size.
